# Among 45 variants in 11 genes, *HDM2* promoter polymorphisms emerge as new candidate biomarker associated with radiation toxicity

**DOI:** 10.1007/s13205-013-0135-3

**Published:** 2013-04-26

**Authors:** Ghazi Alsbeih, Medhat El-Sebaie, Nasser Al-Rajhi, Najla Al-Harbi, Khaled Al-Hadyan, Sara Al-Qahtani, Mohammad Alsubael, Mohammad Al-Shabanah, Belal Moftah

**Affiliations:** 1 Radiation Biology, Biomedical Physics Department, King Faisal Specialist Hospital and Research Centre, MBC-03, P.O. Box 3354, Riyadh, 11211 Saudi Arabia; 2 Radiation Oncology, King Faisal Specialist Hospital and Research Centre, Riyadh, 11211 Saudi Arabia; 3 College of Applied Medical Sciences, King Saud University, Riyadh, 11211 Saudi Arabia

**Keywords:** Single nucleotide polymorphism (SNP), Radiosensitivity, Late reactions to radiotherapy, Fibrosis, Nasopharyngeal carcinoma

## Abstract

Due to individual variations in radiosensitivity, biomarkers are needed to tailor radiation treatment to cancer patients. Since single nucleotide polymorphisms (SNPs) are frequent in human, we hypothesized that SNPs in genes that mitigate the radiation response are associated with radiotoxicity, in particular late complications to radiotherapy and could be used as genetic biomarkers for radiation sensitivity. A total of 155 patients with nasopharyngeal cancer were included in the study. Normal tissue fibrosis was scored using RTOG/EORTC grading system. Eleven candidate genes (*ATM*, *XRCC1*, *XRCC3*, *XRCC4*, *XRCC5*, *PRKDC*, *LIG4*, *TP53*, *HDM2*, *CDKN1A*, *TGFB1*) were selected for their presumed influence on radiosensitivity. Forty-five SNPs (12 primary and 33 neighboring) were genotyped by direct sequencing of genomic DNA. Patients with severe fibrosis (cases, G3–4, *n* = 48) were compared to controls (G0–2, *n* = 107). Results showed statistically significant (*P* < 0.05) association with radiation complications for six SNPs (*ATM* G/A rs1801516, *HDM2* promoter T/G rs2279744 and T/A rs1196333, *XRCC1* G/A rs25487, *XRCC5* T/C rs1051677 and *TGFB1* C/T rs1800469). We conclude that these six SNPs are candidate genetic biomarkers for radiosensitivity in our patients that have cumulative effects as patients with severe fibrosis harbored significantly higher number of risk alleles than the controls (*P* < 0.001). Larger cohort, independent replication of these findings and genome-wide association studies are required to confirm these results in order for SNPs to be used as biomarkers to individualize radiotherapy on genetic basis.

## Introduction

Patients vary considerably in their normal tissue response to radiotherapy (RT) even after identical treatment (Peters 1996; Bentzen and Hendry 1999). These variations can result in severe complications to RT that could compromise the quality of life of cancer survivors. In the era of personalized medicine, biomarkers to predict individual radiosensitivity are being actively sought. This is supported by the demonstration of possible positive therapeutic gains from tailoring the RT dose to the radiosensitivity of each patient (Alsbeih et al. 2003; Tucker et al. 1996; Guirado and Ruiz de Almodovar 2003). Although many treatment-related factors could influence the severity of reactions to RT (Turesson 1990; Bernier et al. 1998), large parts of inter-patient variability is inherent and assumed to emanate from genetic variations between patients (Turesson et al. 1996; Andreassen et al. 2002). The supporting evidence for genetic causes of increased radiosensitivity are the mutations in the ataxia telangiectasis (*ATM*), the *NBS1* (Nijmegen Breakage Syndrome) and the *DNA ligase IV* (*LIG IV*) genes which are components of cell cycle control and DNA repair (Savitsky et al. 1995; Riballo et al. 1999; Varon et al. 1998).

However, gene mutations are rare and can only explain a minority of exquisitely sensitive patients. Therefore, attention was focused on the more common polymorphic variations to explain the wide range of radiosensitivity observed (Andreassen et al. 2002). Genetic variations are frequent in humans, and the challenge of radiogenomic studies is to determine which polymorphisms influence individual radiosensitivity and the risk to develop severe complications following radiotherapy (Parliament 2012). Single nucleotide polymorphism (SNP) is the largest type of inherited genetic variation, of which there are at least 4.5 million (Cargill et al. 1999). The rational is that these polymorphic variations can influence the stability of mRNA, rate of transcription, the protein translation and/or the protein–protein interactions leading to sub-optimal function and expression of different degrees of clinical radiation sensitivity. While molecular investigations attempt to comprehend the mechanisms of action of these small genetic changes and how they interact with host and environmental factors, association studies provide valuable information in determining the degree of linkage between SNPs and radiosensitivity. The hope is to use genetic variations as biomarkers for predictive assays to improve treatment strategies of cancer.

The search for predictive endpoint to tailor the radiation treatment to each individual patient’s radiosensitivity has gone through various phases from cells to gene-based assays. The genetic approach has been boosted by the sequence of the human genome and with the postulation that *all*-*in*-*the*-*genes*, it has the prospect of using genetic variations to predict treatment outcome. This is an attractive approach because these are fixed imprint that can nowadays be determined using DNA extracted from any type of patient’s cells. To identify these variations, many investigators followed an intuitive approach of targeting SNPs in candidate genes arbitrarily involved in radiation response (Andreassen et al. 2003, 2005, 2006a; Alsbeih et al. 2010). Although many studies, carried out often on limited number of RT patients, have reported significant associations, results were globally inconsistent between studies (Parliament and Murray 2010). In addition, a large prospective study has failed to replicate previously reported associations between individual SNP genotype and radiation toxicity (Barnett et al. 2012). However, genome-wide associations study evaluating erectile dysfunction following radiotherapy for prostate cancer has showed significant association not only in a gene that plays a role in male gonad development and function, but also in genes that relate to specific African ancestry that would not have been identified in a cohort of European ancestry (Kerns et al. 2010).

**Fig. 1:**
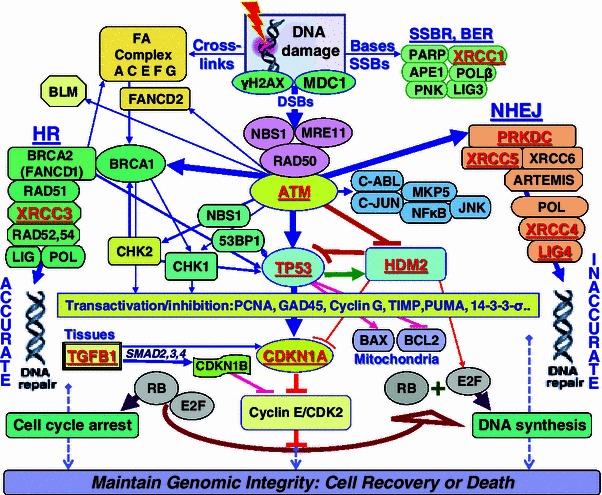
Schematic representation of main pathways involved in response to radiation-induced DNA damage. Base damages (BDs), DNA single-strand breaks (SSBs) and particularly double-strand breaks (DSBs) are the vital lesions produced. BDs and SSBs are efficiently repaired by base-excision (BER) and SSBR mechanisms. DSBs are repaired by two major repair mechanisms, primarily the non-homologous end joining (NHEJ) and secondary the homologous recombination (HR). Radiation-induced damages particularly DSBs, activate panoply of interacting proteins in tissues, cells and mitochondria that lead to the expression and inhibition of hundreds of genes. These results in cell cycle arrest to allow for accurate DNA healing before that the cells enter DNA synthesis with damaged DNA. The aim is to maintain genomic integrity which enables recovery or otherwise triggers cell death. *Lines* represent interactions. *Arrows* indicate activation and *blunt ends* indicate inhibition. *Thickness* represents the strength of the actions. *Underlined font* designates encoding genes selected for this study of genetic polymorphic variations (see text for details)

At the molecular level, ionizing radiation can damage various components in the cells particularly DNA (Fig. 1). Many types of DNA damages are induced including DNA-proteins cross-links, base damages, single and double-strand breaks (SSBs, DSBs). Base damages and SSBs are more frequent and are often efficiently repaired through SSB and base-excision repair mechanisms. DSBs are mainly repaired by two mechanisms, non-homologous end joining (NHEJ) and homologous recombination (HR). Notoriously, DSBs are vital and can activate panoply of downstream molecules leading to cell cycle arrest which allows sufficient time for the DNA to be repaired. Tissular cytokines can also interfere in the processes, and the failure to properly repair damage may trigger cell death through permanent cell cycle arrest or mitochondria-controlled apoptosis. All these signal transduction pathways interplay to ensure maintaining genomic integrity by mediating cell recovery or death. These pathways encompass multitude of genes of which we have selected 11 candidate genes for their presumed or demonstrated influence on radiosensitivity (Andreassen et al. 2003, 2005; Fernet and Hall 2004; Chang-Claude et al. 2005; West et al. 2007; Barnett et al. 2012). These include *CDKN1A* (p21), *TP53*, *ATM*, *HDM2*, *TGFB1*, *XRCC1*, *XRCC3*, *XRCC4*, *XRCC5* (Ku80), *PRKDC*, and *LIG4* which are involved in various pathways (Fig. 1). Since SNPs in these genes implicated in radiation response are likely to affect the outcome to radiation treatment (Parliament and Murray 2010), in this study we have genotyped 45 (12 primary and 33 neighboring) SNPs in 155 head and neck cancer patients treated with definitive radiotherapy, and associated with the grade of fibrosis in normal tissues.

## Materials and methods

### Patients’ population and clinical data

A total of 155 head and neck cancer patients were retrospectively recruited, for this study, during the follow-up of their disease. The patients were treated by definitive RT for nasopharyngeal carcinoma in the Radiation Oncology Section at the King Faisal Specialist Hospital and Research Centre. This cancer site is mainly treated with radiation, with or without chemotherapy, but it does not involve surgery. The treatment was fairly standardized and planned using CT-based (computerized tomography) 3D conformal technique. The upper neck including the primary tumor site (nasopharynx) received the maximum dose through two parallel–opposed–lateral fields using 6 MV photon linear accelerator. Total radiation dose to the upper neck was 66 Gy delivered using 2 Gy per fraction per day over 6.5 weeks. Where possible, patients (*n* = 47) received a boost of two additional fractions to the nasopharynx to bring the dose received to 70 Gy in 7 weeks. In addition, locally advanced stages (II–IVB, *n* = 74) received neoadjuvant and concurrent chemotherapy consisting of cisplatinum and epirubicin (Al-Amro et al. 2005). The grade (G) of subcutaneous and deep tissue fibrosis, a late radiation-induced complication, was jointly scored by two participating physicians at the recruitment visit according to the RTOG/EORTC grading system. For group comparison, patients with major toxicity (Cox et al. 1995), severe fibrosis (G3–4), were referred to as the radiosensitive group (cases, *n* = 48) and were compared to patients with minor (G0–2) fibrotic reactions (controls, *n* = 107). The Institutional Review Board (IRB) had approved the study and all patients had signed informed consent.

### DNA extraction, amplification, sequencing and genotyping of polymorphisms

During the regular follow-up of the patients, a 5-ml blood sample was drawn and/or 3 mm punch skin biopsy was taken from consenting patients. Where applicable, fibroblast culture was established using standard protocol (Torres et al. 2004). DNA was extracted from blood or cultured fibroblasts using the appropriate puregene DNA purification kit (Gentra System, USA) according to the manufacturer’s instruction. The selected 12 primary SNPs along with the PCR primers are listed in Table 1. Relevant segments of DNA were amplified by thermal cycling (95 °C for 15 min, 39 rounds of 95 °C for 1 min, 56 °C for 1 min and 72 °C for 1 min and final extension at 72 °C for 7 min) using HotStarTaq DNA polymerase (Qiagen), and 50 ng template DNA in 25 μm volume with standard reaction conditions. The amplified fragment was directly sequenced using the DYEnamic ET Dye terminator cycle sequencing kit (Amersham Biosciences) according to the manufacturer’s instruction, and were run on the MegaBase 1,000 sequencer (Applied Biosystems). Sequencing results were aligned to the corresponding reference sequence and the primary SNPs, along with neighboring SNPs that are in the sequenced fragments, were genotyped using SeqManII sequence analysis software (DNASTAR Inc.).

**Table 1:** Primary SNPs assessed and primers used for PCR amplification and DNA sequencing

Gene	Codon	Base change	Amino acid change	PCR primers		NCBI dbSNP id/Ref.
				Forward	Reverse	
*CDKN1A (p21, Cip1)*	31	C/A	Ser/Arg	CGCCATGTCAGAACCGGCT	TTCCATCGCTCACGGGCC	rs1801270
*TP53 (p53)*	72	G/C	Arg/Pro	TGGTCCTCTGACTGCTCTTTT	AACTGACCGTGCAAGTCACA	rs1042522
*HDM2 (MDM2)*	Promoter	Position 309	–	TTTGGGGGTCTTCTGGTAAA	TCCTAACTCTGATATCCCAAG	rs2279744
*ATM*	1853	G/A	Asp/Asn	ATATGTCAACGGGGCATGAA	CATTAATATTGCCAGTGCAAG	rs1801516
*XRCC1*	399	G/A	Arg/Gln	GCCCCTCAGATCACACCTAA	GATAAGCAGGCTTCACAGAGC	rs25487
*XRCC3*	241	C/T	Thr/Met	GGTTAGGCACAGGCTGCTAC	CTTGCTGACCAGCATAGACAA	rs861539
*XRCC4*	247	G/T	Ala/Ser	GCTTACTGATAAATCTGCTGCCTA	TGTATGAATGCTTGCTCACACT	rs3734091
*XRCC5 (Ku80)*	3′ UTR	A/G	–	CAAGGGATAATTTAGACCCCATA	GGGCCAAAAGGTCTTTTCTT	rs1051685
*LIG4 (DNA Ligase IV)*	591	A/G	Ile/Val	CCCTGGACGACCTAGAACAA	GGAGAGCAATCCCAGGAATA	rs2232641
*LIG4 (DNA Ligase IV)*	9	C/T	Thr/lle	TCAAATTAGGGTTGGAGCAAA	TTCCATAGGCCATTCTCTCTC	rs1805388
*PRKDC (DNA*-*PKcs)*	3434	A/G	Ile/Thr	CCTTCCATTAGAGTGCCAT	ATGCACTGCACACACTAACG	rs7830743
*TGFB1 (TGFβ1)*	10	C/T	Leu/Pro	AGCCTCCCCTCCACCACT	TGGGTTTCCACCATTAGCAC	rs1982073

### Data analysis

The association between SNP allelic frequencies and grade of fibrosis were measured by the odds ratio (OR) with its 95 % confidence interval. Significance of OR was assessed by the Chi-square (*χ*^2^) test. In case the latter was not applicable, the Fisher’s exact test was used. A *P* value of 0.05 or less is considered statistically significant. The alleles showing statistically significant (*P* ≤ 0.05) association with increased clinical radiosensitivity were considered as risk allele and given a score of one. Therefore, patients homozygous for a risk allele have a score of two, heterozygous have a score of one while patients who do not harbor the risk allele have a score of zero. The number of risk alleles for each patient was calculated by summing the scores of the different SNPs significantly associated with radiosensitivity. Difference between groups was assessed by the non-parametric Mann–Whitney rank sum test. Correction for multiple comparisons was carried out using Bonferroni method, which indicates statistical significance when the *P* value is lower than the type I error (0.05) divided by the number of comparisons. Statistical analysis was carried out using the SigmaPlot platform (Version 12.0, SPSS Science, IL, USA) and the free online softwares, VassarStats: Website for Statistical Computation, Vassar College, Poughkeepsie, NY, USA (http://faculty.vassar.edu/lowry/odds2x2.html) and Case Control Studies: Tests for Association, Institute of Human Genetics, Helmholtz Center Munich, Germany (http://ihg.gsf.de/cgi-bin/hw/hwa1.pl).

## Results

### Patients and treatment

The age of patients at RT ranged between 15 and 77 years/old with a median of 47. There were 39 females and 116 males. All patients had completed at least 24 months of follow-up (range 24–180 months, median 40 months). Acute reactions such as erythema, dermatitis and mucositis, were available for 62 patients only that were retrieved from medical charts and have not been analyzed due to small number. Late normal tissue reactions to radiotherapy (xerostomia, skin atrophy and subcutaneous and deep tissue fibrosis) were scored by two participating physicians during the follow-up visit of the patients. Only grade of fibrosis is reported here because it was completed for all patients. There were 17, 54, 36, 38, and 10 patients who had exhibited fibrotic reactions of grade 0, 1, 2, 3 and 4, respectively. Patients classified as having major toxicity (G3 and G4, cases) were compared to those having minor reactions (G0, G1 and G2, controls) (Cox et al. 1995). Therefore, patients with severe subcutaneous and/or deep tissue fibrosis (G3–4, cases, *n* = 48) were referred to as radiosensitive and were compared to the remaining patients having no, mild or moderate fibrosis (G0–3, controls, *n* = 107). The distribution of controls and radiosensitive patients according to chemotherapy and radiation boost received were comparable. Briefly, 79 and 54 patients had received chemotherapy and RT boost; respectively, who were proportionally distributed between controls and cases. Thus, the ratio of patients who received chemotherapy to the patients who did not were comparable in the control and the radiosensitive groups (0.50 vs. 0.52, *P* = 0.80). Similarly, the average total doses received (with and without boost) in controls (67.50 Gy, SD = 1.94) and in the radiosensitive groups (67.17 Gy, SD = 1.84) were not significantly different (*P* = 0.35).

### Genotyping analysis

**Fig. 2:**
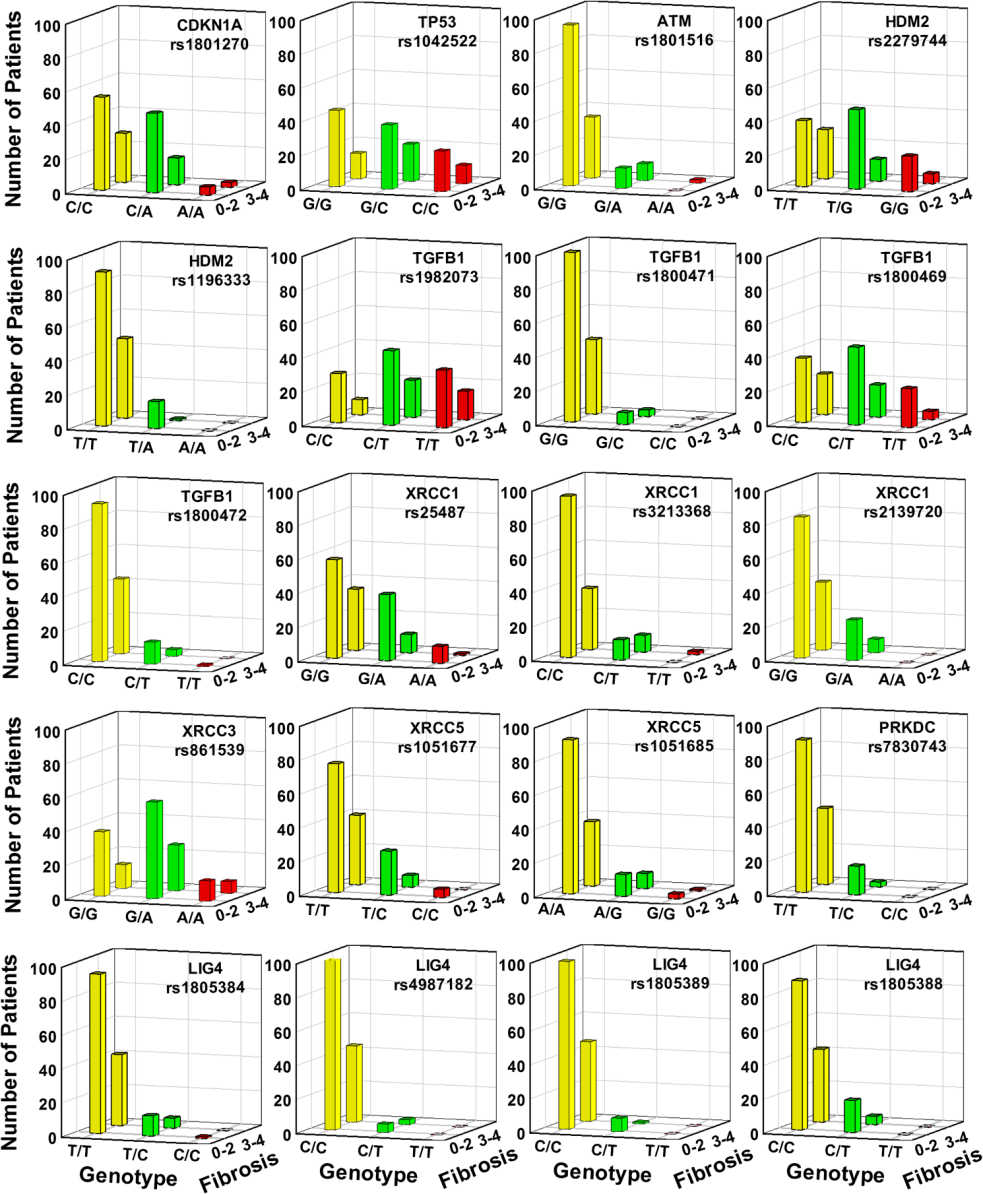
Genotypes’ distribution of 20 SNPs that showed five or more individuals with minor alleles in 155 nasopharyngeal cancer patients who developed minimal (0–2) or severe (3–4) grade of radiation-induced fibrosis

A total of 45 SNPs were genotyped. These were detected in 12 DNA fragments of 11 genes (1 in *CDKN1A*; 2 in *TP53*, *ATM*, *PRKDC*, *XRCC4*; 3 in *HDM2*; 4 in *XRCC3*, *XRCC5*; 5 in *XRCC1*; 8 in *LIG4*; 12 in *TGFB1*). In numbers, 15 SNPs were all wild types and 10 SNPs showed 1 or 2 variant genotype. There were 20 SNPs having variant genotypes frequency >2; the distribution of which in relation to late radiotoxicity (grade of fibrosis) is depicted in Fig. 2. There were wide variations in the distribution of the different genotypes according to the grade of fibrosis.

The allelic frequencies of the 45 assessed SNPs are given in Table 2. Comparison between cases and controls revealed statistically significant association (*P* < 0.05) for six SNPs (*ATM* rs1801516, *HDM2* rs2279744, *HDM2* rs1196333, *TGFB1* rs1800469, *XRCC1* rs25487 and *XRCC5* rs1051677). Interestingly, apart from *ATM* where the variant *A* allele was associated with increased risk, the variant alleles of the remaining significantly associated SNPs showed decreased risk (odds or risk ratios <1) to develop severe fibrosis, and therefore, they exhibit protective effect. The association observed for *ATM* rs1801516, *HDM2* rs2279744, *XRCC1* rs25487 remains statistically significant after taking into consideration multiple comparisons using Bonferroni correction.

**Table 2:** Allele frequencies of the assessed polymorphisms in 155 head and neck cancer patients who either developed minimal (controls: G0–2) or severe (cases: G3–4) late reactions (fibrosis) after radiotherapy

Gene and SNP	Allele 1^a^		Allele 2^b^		Odds ratio	*P* value
	Cases	Controls	Cases	Controls	(95 % CI)	
	*n* (%)	*n* (%)	*n* (%)	*n* (%)		
*CDKN1A* C/A rs1801270	74 (77)	157 (73)	22 (23)	57 (27)	0.82 (0.47–1.44)	0.49
*TP53* G/C rs1042522	52 (54)	112 (52)	44 (46)	102 (48)	0.93 (0.57–1.51)	0.76
*TP53* C/T rs1800371	96 (100)	213 (99.5)	0 (0)	1 (0.5)	0	1.00*
*ATM* G/A rs1801516	82 (85)	202 (94)	14 (15)	12 (6)	2.86 (1.18–6.48)	**<0.01**
*ATM* A/T rs1801673	96 (100)	214 (100)	0 (0)	0 (0)	–	–
*HDM2* G/C rs7484572	96 (100)	214 (100)	0 (0)	0 (0)	–	–
*HDM2* T/G rs2279744	71 (74)	125 (58)	25 (26)	89 (42)	0.49 (0.29–0.84)	**<0.01**
*HDM2* T/A rs1196333	95 (99)	198 (93)	1 (1)	16 (7)	0.13 (0.02–0.99)	**0.02**
*TGFB1* G/A rs9282871	95 (99)	213 (99.5)	1 (1)	1 (0.5)	2.24 (0.14–36.23)	1.00*
*TGFB1* C/T rs1982073	40 (42)	102 (48)	56 (58)	112 (52)	1.28 (0.78–2.07)	0.32
*TGFB1* G/C rs1800471	92 (96)	207 (97)	4 (4)	7 (3)	1.28 (0.37–4.50)	0.74*
*TGFB1* C/T rs4987025	96 (100)	214 (100)	0 (0)	0 (0)	–	
*TGFB1* C/T rs1800469	67 (70)	122 (57)	29 (30)	92 (43)	0.57 (0.34–0.96)	**0.03**
*TGFB1* G/A rs11466314	96 (100)	213 (99.5)	0 (0)	1 (0.5)	0*	1.00*
*TGFB1* C/T rs35318502	96 (100)	214 (100)	0 (0)	0 (0)	–	–
*TGFB1* del rs8179182	94 (98)	214 (100)	2 (2)	0 (0)	3.28** (2.77–3.88)	0.10*
*TGFB1* C/A rs35383147	96 (100)	214 (100)	0 (0)	0 (0)	–	–
*TGFB1* ins rs34233206	96 (100)	214 (100)	0 (0)	0 (0)	–	–
*TGFB1* C/T rs1800472	92 (96)	199 (93)	4 (4)	15 (7)	0.58 (0.19–1.79)	0.33
*TGFB1* C/T rs11466334	95 (99)	213 (99.5)	1 (1)	1 (0.5)	2.24 (0.14–36.22)	1.00*
*XRCC1* G/A rs2271980	96 (100)	214 (100)	0 (0)	0 (0)	–	–
*XRCC1* G/A rs25487	83 (86)	155 (72)	13 (14)	59 (28)	0.41 (0.21–0.79)	**<0.01**
*XRCC1* C/T rs3213368	87 (91)	193 (90)	9 (9)	21 (10)	0.95 (0.42–2.16)	0.90
*XRCC1* G/A rs2139720	88 (92)	190 (89)	8 (8)	24 (11)	0.72 (0.31–1.67)	0.44
*XRCC1* C/T rs3213369	96 (100)	213 (99.5)	0 (0)	1 (0.5)	0	1.00*
*XRCC3* G/A rs41285494	96 (100)	214 (100)	0 (0)	0 (0)	–	–
*XRCC3* G/A rs861539	55 (57)	133 (62)	41 (43)	81 (38)	1.22 (0.75–1.99)	0.42
*XRCC3* A/C rs3212112	95 (99)	213 (99.5)	1 (1)	1 (0.5)	2.24 (0.14–36.22)	1.00*
*XRCC3* C/T rs3212113	96 (100)	214 (100)	0 (0)	0 (0)	–	–
*XRCC4* A/C rs2974446	96 (100)	214 (100)	0 (0)	0 (0)	–	–
*XRCC4* G/T rs3734091	95 (99)	213 (99.5)	1 (1)	1 (0.5)	2.24 (0.14–36.23)	1.00*
*XRCC5* A/G rs41296835	96 (100)	213 (99.5)	0 (0)	1 (0.5)	0	1.00*
*XRCC5* T/C rs1051677	89 (93)	178 (83)	7 (7)	36 (17)	0.39 (0.17–0.91)	**0.02**
*XRCC5* G/T rs41437350	96 (100)	214 (100)	0 (0)	0 (0)	–	**–**
*XRCC5* A/G rs1051685	85 (89)	195 (91)	11 (11)	19 (9)	1.33 (0.61–2.91)	0.48
*PRKDC* T/C rs7830743	93 (97)	197 (92)	3 (3)	17 (8)	0.37 (0.11–1.31)	0.11
*PRKDC* A/G rs8178228	96 (100)	214 (100)	0 (0)	0 (0)	–	–
*LIG4* T/C rs1805384	90 (94)	200 (93)	6 (6)	14 (7)	0.95 (0.36–2.56)	0.92
*LIG4* C/A rs1805383	96 (100)	214 (100)	0 (0)	0 (0)	–	–
*LIG4* C/T rs4987182	93 (97)	209 (98)	3 (3)	5 (2)	1.35 (0.32–5.76)	0.71*
*LIG4* C/T rs1805389	95 (99)	206 (96)	1 (1)	8 (4)	0.27 (0.03–2.20)	0.28*
*LIG4* C/T rs1805388	91 (95)	195 (91)	5 (5)	19 (9)	0.56 (0.20–1.56)	0.26
*LIG4* G/A rs2232636	96 (100)	214 (100)	0 (0)	0 (0)	–	–
*LIG4* G/A rs2232641	96 (100)	214 (100)	0 (0)	0 (0)	–	–
*LIG4* A/G rs3093766	96 (100)	214 (100)	0 (0)	0 (0)	–	–

Significantly associated SNPs are highlighted in bold

* *P* value represents the 2-tailed Fisher’s exact test, calculated in case the Chi-square cannot be determined, ** risk ratio (RR) is calculated when odds ratio (OR) is inaccurate

^a^*Allele 1*: majority or wild-type allele

^b^*Allele 2*: minority or variant allele

### Influence of the number of risk alleles on the grade of fibrosis

**Fig. 3:**
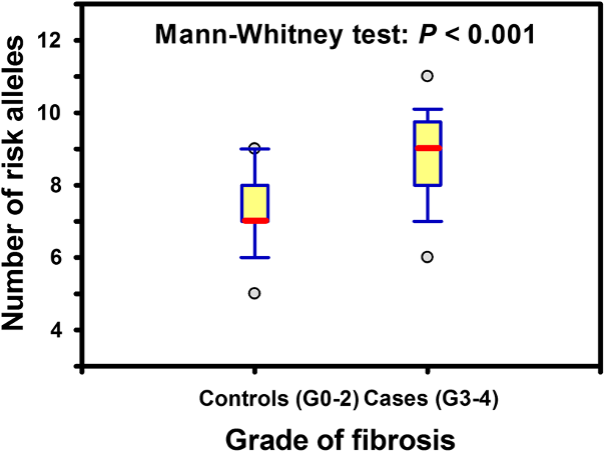
Box plot analysis of the relationship between the number of risk alleles and clinical radiosensitivity of the two groups of cancer patients who either developed minimal (G0–2) or severe (G3–4) fibrotic reaction. *Bold lines* indicate the median number of risk alleles. *Upper* and *lower* boundaries of *boxes* indicate the 75th and 25th percentile. *Barsabove* and *below boxes* indicate the 90th and 10th percentiles. Data points represent outliers

The alleles that showed statistically significant associations with increased risk to develop severe fibrosis (the variant allele for *ATM* rs1801516 and the majority or wild-type alleles of *HDM2* rs2279744, *HDM2* rs1196333, *TGFB1* rs1800469, *XRCC1* rs25487 and *XRCC5* rs1051677) have been counted to calculate the number of risk alleles for each patient (see “Data analysis”). The number of risk alleles ranged between 3 and 10 (median = 7) in controls compared to 5–11 (median = 9) in the radiosensitive patients. The relationship between the number of risk alleles and clinical radiosensitivity (G0–2 compared to G3–4) has been analyzed by box plot (Fig. 3). Although variations were present, patients who developed severe fibrosis (G3–4) showed a clear trend to harbor higher number of risk alleles. The comparison between the two groups showed a statistically significant difference in the median number of risk alleles between cases and controls (Mann–Whitney test, *P* < 0.001).

## Discussion

The aim of this study was to evaluate in our local cancer patients whether genetic polymorphic variations in candidate genes involved in radiation response mediated through cell cycle control and DNA repair mechanisms (Fig. 1) are associated with the severity of RT-induced fibrotic reactions in normal tissues. The 155 head and neck cancer patients included in this report had nasopharyngeal carcinoma. This cancer site is prevalent in Saudi Arabia and is ideal for this type of study because patients follow standardized treatment with curative radiation without surgery. Radiotherapy is the main treatment and it was delivered using 6 MV photon linear accelerator. The standard cumulative radiation dose to the upper neck, where radiation effects are scored, was 66 Gy given as 2 Gy per fraction. A boost of two fractions to the nasopharynx, which bring the total dose to 70 Gy, was given to certain patients taking into consideration the stage of the disease and the judgment of the treating physician. Locally advanced tumors are also treated with neoadjuvant and concurrent chemotherapy consisting of cisplatinum and epirubicin (Al-Amro et al. 2005).

Patients were enrolled in the study during the follow-up of their disease. Only those who completed at least 2 years of follow-up were reported here. This is in principle sufficient for the appearance and the intensification of late radiotoxicity (Barnett et al. 2012). Although various end points of acute and late complications following radiotherapy were scored for each patient, data on subcutaneous and deep tissues fibrosis were completed for all 155 reported patients. Associated diseases were uncommon: 15 patients had diabetes (10 in controls and 5 in cases), 7 were hypertensive (3 in controls and 4 in cases), 2 patients with systemic lupus erythematous (controls) and 1 patient had scleroderma with severe Raynaud’s phenomena (control). Thus, the associated diseases do not account for the observed differences in radiotoxicity in this cohort.

To maximize chances of seeing differences, patients with severe subcutaneous or deep tissue fibrosis (G3–4, cases, *n* = 48) were compared to patients with minimal to moderate fibrosis (G0–2, controls, *n* = 107). This classification is slightly different from that reported in earlier where G0–1 group was compared to G2–3 because there was no G4 in that pilot study (Alsbeih et al. 2010). Treatment characteristics were comparable between the radiosensitive cases and the controls; taking into account the total radiation dose and the chemotherapy received. Thus, overall no differences could be attributed to associated diseases or treatment-related factors.

The 12 candidate primary polymorphisms included in this study (Table 1) were selected based on previous reports on the potential association between radiosensitivity and SNPs (Andreassen et al. 2002; Alsbeih et al. 2010; Barnett et al. 2012) or radiation-induced levels of the encoded protein (Alsbeih et al. 2009a). Since we have used direct DNA sequencing technique, it was possible to genotype neighboring SNPs. This allowed genotyping of 45 SNPs in the 11 candidate genes (Table 2). These genetic variations were either synonymous, nonsynonymous, insertion or deletion that may have impact on protein level and contribute to variations between patients.

Among the 45 genetic variations scored, six SNPs (*ATM* rs1801516*, HDM2* rs2279744, *HDM2* rs1196333, *TGFB1* rs1800469, *XRCC1* rs25487 and *XRCC5* rs1051677) showed significant association (*P* < 0.05) between allelic frequency and grade of fibrosis following RT (Table 2). Moreover, the association found for *ATM* rs1801516*, HDM2* rs2279744 and *XRCC1* rs25487 remained statistically significant after taking into consideration Bonferroni’s multiple comparisons correction. As compared to the controls (G0–2), the radiosensitive group (G3–4) harbored relatively higher number of variant *ATM* rs1801516 *A* allele which appeared to be a risk factor (OR = 2.86, CI 95 % 1.18–6.48, *P* < 0.01), and lower numbers of the variants *HDM2 rs2279744 G* (OR = 0.49, CI 95 % 0.29–0.84, *P* < 0.01)*, HDM2 rs1196333 A* (OR = 0.13, CI 95 % 0.02–0.99, *P* = 0.02)*, TGFB1 rs1800469 T* (OR = 0.57, CI 95 % 0.34–0.96, *P* = 0.03), *XRCC1* rs25487 *A* (OR = 0.41, CI 95 % 0.21–0.79, *P* < 0.01), and *XRCC5* rs1051677 *C* (OR = 0.39, CI 95 % 0.17–0.91, *P* = 0.02) alleles which appeared to have protective effect; therefore, the wild-type alleles were the risk factors. These are interesting results that plead in favor of the potential use of genetic markers as predictors of normal tissue response, particularly that the subject is a hot topic debate (Barnett et al. 2012).

To our knowledge, this is the first study on the association between *HDM2* T309G promoter (rs2279744) and radiosensitivity; previous studies were only concerned with its cancer predisposing potential (Bond et al. 2005; Sun et al. 2010; Al-Hadyan et al. 2012). The *HDM2* gene encodes a protein that is a key component of TP53 protein signaling pathway (Fig. 1). *HDM2* is transcriptionally activated by TP53 (Barak et al. 1993). It regulates the function of TP53 in several ways including the ubiquitin E3 ligase for TP53 and targets its degradation by the proteasomal pathway (Honda et al. 1997); *HDM2* is responsible for the nuclear to cytoplasmic shuttling of TP53, thus inhibiting its function as a transcription factor (Roth et al. 1998). *HDM2* also binds TP53 and inhibits transactivation (Momand et al. 1992). Although mutations in *HDM2* are infrequent (Tamborini et al. 2001), *HDM2* protein is overexpressed in about 5–10 % of human tumors (Ladanyi et al. 1993). In addition, *HDM2* protein interacts with the S phase-promoting factor, E2F1, and increases its function (Martin et al. 1995). These two proteins were also found to interact with a number of heterogeneous nuclear ribonucleoproteins (hnRNP), which orchestrate mRNA processing in response to ionizing radiation (Haley et al. 2009).

The functional polymorphic variant in the *HDM2* promoter at position 309 (rs2279744) have been suggested to affect the transcriptional activator SP1 binding, thereby modulating *HDM2* transcription level. The *G* variant has been shown to increase the affinity for Sp1, resulting in higher levels of *HDM2* mRNA and protein and the subsequent attenuation of the TP53 pathway (Bond et al. 2004). The impact of this genetic variation on *HDM2* levels have a snow-balling effect on TP53 amounts in the cell, and the *G* allele which leads to higher *HDM2* transcription was shown to attenuate the TP53 response which could alter cellular response to radiation therapy and DNA-damaging drugs (Nayak et al. 2007). Results presented here showed that the same variant *G* allele, and also the variant *G* allele in the neighboring *HDM2* rs2279744 SNP, is associated with reduced risk to develop late normal tissues complications, a phenomenon that is dependent on the amount of cell depletion following radiotherapy. Therefore, in line with our results, it is conceivable that this *HDM2 G* variant allele could promote cell survival following irradiation and thus, cells would appear more radioresistant, despite the probable high risk of genomic instability due to presumably attenuated TP53 (Fig. 1). This may also have implication for the promotion of secondary cancers following radiotherapy.

This is also the first study to report association between *XRCC5* (KU80) polymorphisms and clinical radiosensitivity. XRCC5 is a component of the non-homologous end joining (NHEJ) to repair DNA double-strand breaks (Fig. 1). Previously, SNPs in *XRCC5* have been shown to influence cancer risk and chromosomal radiosensitivity (Willems et al. 2008; Al-Hadyan et al. 2012). Our study showed that, although uncommon, the variant *XRCC5* rs1051677 *C* allele was more frequent in the controls (Fig. 2), thus it has a protective effect.

As reported previously, the variant *ATM* rs1801516 *A* allele (Asn) was significantly associated with increased radiation sensitivity (Andreassen 2005; Alsbeih et al. 2007b). Other studies have also shown similar association with enhanced risk of various adverse reactions after RT for breast and prostate cancer (Angele et al. 2003; Hall et al. 1998; Andreassen et al. 2006b). In contrast, the wild type or majority *XRCC1* rs25487 allele (Arg) was associated with increased risk to develop late reactions to radiotherapy (reviewed in Andreassen 2005). This suggests that the variant (or minority) allele could confer higher radioresistance in favor of normal tissues involved in the radiation treatment. The XRCC1 protein is required for efficient DNA single-strand breaks repair to maintain genomic stability (Fig. 1). Its reduction leads to increased sensitivity to cell killing by ionizing radiation (Brem and Hall 2005). Although the codon 399 is situated in the BRCT I active domain of the protein, both wild type and variant alleles were found to be in vitro equally functional (Taylor et al. 2002). The results of present and similar clinical studies seem to be counter intuitive to in vitro studies; however, a study by Brem et al. (2006) suggested that it is the haplotype in the *XRCC1* gene (i.e., segregation with other SNPs) rather than the G28152A SNP per se that is associated with cellular or clinical radiosensitivity.

*TGFB1* encodes for the versatile cytokine TGFB1 which is assumed to be involved in the tissular modulation of inflammation in response to tissue injuries (Fig. 1). Therefore, SNPs that can modulate protein production can result in excessive deposition of scar tissue and fibrosis (Border and Noble 1994). Therefore, many SNPs have been studied in the literature. Between 12 neighboring *TGFB1* polymorphisms, the significantly associated SNP rs1800469 seems to be different from the rs1982073 reported previously (Alsbeih et al. 2010), but in agreement with other studies (Andreassen et al. 2003; Azria et al. 2008; Giotopoulos et al. 2007; Quarmby et al. 2003). Thus, the effect of haplotype needs to be clarified as co-segregation of polymorphic variations in *TGFB1* gene has been suggested to play a role in radiation response. De Ruyck et al. (2006) have reported that three different variations in *TGFB1* were associated with the risk of developing late severe reactions after gynecologic RT, where analysis revealed two major haplotypes but could not distinguish radiosensitive from nonradiosensitive patients.

This study, however, did not show significant association for SNPs in *CDKN1A*, *TP53*, *LIG IV*, *PRKDC*, *XRCC3* and *XRCC4*. These negative results, however, do not negate the importance of these genes to radiosensitivity as mutations in *TP53* and *LIG IV* are well-known example of genetic disorder with potential impact on radiosensitivity. In addition, an association between *TP53* G72T and in vitro cellular radiosensitivity was reported (Alsbeih et al. 2007a). This strengthens the widely held belief that the correlation between cellular and clinical radiosensitivity is somewhat weak and overwhelmed with multitude of tissues and patient-related factors. In addition, in a large independent dataset Barnett et al. (2012) also could not validate previously reported associations between genotype and radiation toxicity. Furthermore, our results do not exclude other genetic variations in these genes and larger studies are required to unravel the influence of subtle genetic changes on radiation response.

The risk alleles associated with increased clinical radiosensitivity were either variant or wild type (Table 1). This indicates that not all variant SNPs are risky. From an evolutionary perspective, it is possible that the substitutions observed frequently are likely to be neutral or favorable, whereas those observed rarely are likely to be deleterious (Zhu et al. 2004). More importantly, group comparison between cases and controls showed statistically significant difference in the median number of risk alleles (*P* < 0.001) with the radiosensitive group (G3–4) harboring higher number of risk alleles (Fig. 3). This is an important demonstration of the combined effect of different genetic variations and supports the assumption that radiosensitivity is a complex genetic trait. Therefore, harboring higher number of risk alleles has incremental effect on complications to radiotherapy. This illustrates that radiation response requires the concerted action of multiple genes and, therefore, it is a complex genetically controlled trait with the outcome being determined by multitude of additive effects. This conclusion is further substantiated by the assumption that the combined risk alleles effect on radiosensitivity may also incorporate variations in mitochondrial DNA, the energy producing cytoplasmic organelles, as a subset of patients of this study have also showed association with genetic variations in mtDNA (Alsbeih et al. 2009b). The genomic revolution with the advent of high-throughput techniques can help uncovering the panoply of these interacting factors at the DNA (genome), RNA (transcriptome) or protein (proteome) level. Research using genome-wide analysis tools heralds the future of individualized radiation treatment in broadly personalized medicine. In addition to predictive testing, the identified genes and their products could become targets for innovative therapies in radiosensitive individuals.

## Conclusions

Between 45 SNPs in 11 genes involved in cell cycle control and DNA repair, 6 showed significant association with radiation toxicity in Saudi radiotherapy patients. Although many of these SNPs were studied before with variable results, this is the first study to include SNPs in *HDM2* gene where two SNPs in the promoter region were significantly associated with fibrotic reaction. In addition, the radiosensitive patients harbored significantly higher number of risk alleles than the controls (*P* < 0.001). Larger cohort, independent replication of these findings and genome wide association studies (GWAS) are required to confirm these results and validate the use of SNPs as biomarkers to individualize radiotherapy on genetic basis.
